# An online intervention targeting intimate partner violence in perinatal women with recent mental health care utilization: a multisite randomized clinical trial

**DOI:** 10.1007/s00737-026-01703-4

**Published:** 2026-04-23

**Authors:** Dawn M. Johnson, Ananda Sen, Dongru Chen, Kristina Countryman, Maria Muzik, Briana Joseph, Golfo Tzilos Wernette, Caron Zlotnick

**Affiliations:** 1 University of Akron, Akron, USA; 2 University of Michigan, Ann Arbor, USA; 3 Women & Infants Hospital, Providence, USA; 4 University of Cape Town, Cape Town, South Africa

## Abstract

**Purpose:**

This study evaluated the efficacy of an online intervention targeting intimate partner violence (IPV) in perinatal women, *S*trength for *U* in *R*elationship *E*mpowerment (SURE), relative to an attention, time, and information-matched control, on IPV severity, positive affect and well-being, and perceived emotional support across a 12-month follow-up.

**Methods:**

Perinatal women (*N* = 122) who reported past 12-month IPV and recently engaged in mental health treatment were randomized to SURE (*n* = 65) or control (*n* = 57). SURE participants received a 40-minute online intervention based in motivational interviewing and empowerment. Control participants viewed a 40-minute online video of popular entertainment, followed by questions about preferences. Both conditions also included a brief 10-15-minute telephone-delivered booster session one month after the online session. Follow-up assessments occurred at 6 weeks, and 3-, 6-, and 12-months since the baseline visit.

**Results:**

Both interventions exhibited a significant drop in IPV severity from baseline to 12-month follow-up, with no significant differences between arms (range of standardized effect sizes for the time by group interaction *=* 0.20–1.65). Significant intervention effects were found for both positive affect and well-being (range of standardized effect sizes for the time by group interaction = -0.04-1.62) and perceived emotional support (range of standardized effect sizes for the time by group interaction = 0.65–2.10), where participants in SURE exhibited significant increases in both outcomes, while control participants did not.

**Conclusion:**

Results suggest that SURE provides a good model for an online intervention for perinatal women who have sought mental health services.

## Highlights

- A study of perinatal women with intimate partner violence compared the efficacy of a brief online intervention to a control condition.
- Those in the intervention reported gains in positive affect and well-being, and perceived emotional support compared to the controls.
- Both groups reported reductions in intimate partner violence (IPV) severity from baseline and across follow-up.
- The study suggested that online interventions can be delivered safely and may be beneficial for perinatal women with IPV.

## Introduction

Intimate partner violence (IPV) is a serious public health problem in which the perinatal period is a particularly vulnerable time. A recent meta-analysis reports a pooled IPV prevalence of 12.8% during pregnancy and 14.7% during the post-partum period (Chen et al. 2024). Additionally, a systematic review (Adhikari et al. 2025) found a sample-weighted average prevalence of physical perinatal IPV (P-IPV) of 14.7%. Studies have reported rates of sexual P-IPV up to 20% (Hahn et al. 2018) and 38.3% for psychological P-IPV (Mohammadi et al. 2025), with rates varying as a function of population and methodology. P-IPV is associated with significant maternal morbidity, with a recent literature review concluding that P-IPV has adverse effects on the mother (e.g., mental health difficulties), developing fetus (e.g., fetal injury/death), and the child (e.g., behavioral/emotional problems; Agarwal et al. 2023). Importantly, 45.3% of pregnancy-associated homicides are IPV-associated (Palladino et al. 2011). Thus, there is a particular need to target IPV in this vulnerable population (Stewart et al. 2017). Few interventions, however, have demonstrated efficacy. While only perpetrators have control over their violent behavior, women who experience IPV can be empowered to access resources and seek support, which can increase their health and well-being and decrease their risk for revictimization. Perinatal women who recently utilized mental health treatment are especially at risk for IPV and IPV-related consequences, given the strong relationships between IPV and multiple psychiatric disorders (Lou et al. 2024; White et al. 2024; Reyes et al. 2023) and the high rates of IPV in mental health treatment settings relative to other medical settings (e.g., antenatal clinics; Rees et al. 2011). Despite this need, research finds low rates of IPV screening and intervention in mental health settings (Chang et al. 2011), and no evidence-based interventions exist that target IPV in mental health treatment-seeking perinatal women.

Online interventions are self-directed, unrestricted by time or place, and low-cost, with several important strengths for working with perinatal women who have experienced IPV (Carrandi et al. 2023). The use of technology for IPV screening has demonstrated acceptability, feasibility, and improved detection (Klevens et al. 2012; Rhodes et al. 2002). Online interventions overcome numerous obstacles to IPV intervention (e.g., transportation; Hasselle et al. 2020; Hui et al. 2023) and are highly acceptable to perinatal women (Van Den Heuvel et al. 2018); however, no evidence-based online interventions exist that address the unique needs of perinatal women who have experienced IPV and recently utilized mental health treatment (Jahanfar et al. 2014; Hameed et al. 2021).

*S*trength for *U* in *R*elationship *E*mpowerment (SURE) is an innovative, low-cost online intervention designed to address known barriers to early IPV intervention (Hasselle et al. 2020) and be easily integrated into clinical care. Results from a pilot randomized controlled trial (RCT) support SURE’s acceptability and preliminary efficacy (Zlotnick et al. 2019). The purpose of this study was to evaluate the efficacy of SURE, compared to an attention, time, and information-matched control condition. Our *primary aim* was to test the hypothesis that SURE, relative to control, is associated with lower IPV severity at 6-week, 3-, 6-, and 12-month follow-up. Our *secondary aim* was to test the hypotheses that SURE, compared to control, will result in greater (a) positive affect and well-being and (b) perceived emotional support across the 12-month follow-up.

## Materials and methods

A detailed description of the study methods, a priori power analyses and target sample size, both intervention conditions (Johnson et al. 2020a), and safety protocols implemented during uthor COVID (Johnson et al. 2022) are provided elsewhere. This study was initiated before the normative practice of data depositories; participants did not consent to have their data publicly available. Data are available by request to the last author.

### Participants

Participants were 122 perinatal women, 18 to 45 years old, who had recently sought mental health treatment. Inclusion criteria were: (1) pregnant or up to 12^1^ months postpartum, (2) IPV positive screen in the last 12 months as measured by the Woman Abuse Screening Tool (WAST; Brown et al. 2000); (3) sought mental health treatment while pregnant or within 12 months postpartum,^2^ (4) able to understand study materials in English, and (5) willing to complete all intervention and assessment sessions.

### Intervention conditions

Participants were randomized to *SURE* (*n* = 65) or control (*n* = 57). SURE is a 40-minute trauma-informed (SAMHSA, 2014) online intervention based in motivational interviewing (Miller and Rollnick 2012), which aligns with the empowerment-based approach recommended for IPV (Herman 2015). SURE uses the Computerized Intervention Authoring System (CIAS) software (Ondersma et al. 2007), which supports the development of highly interactive online interventions. Participants receive personalized feedback regarding the types of IPV they endorsed, education regarding types of IPV, associated risks of IPV on the woman, fetus, child, and mental and physical consequences of IPV, and risks associated with untreated mental health issues, emphasizing the bidirectional relationship between IPV and mental health. Participants’ readiness to utilize IPV and mental health resources is assessed, and intervention is tailored to their readiness. Participants are offered a menu of choices for when, why, and how they would like to enact change, as well as an option to create a personalized safety plan. The importance of formal and informal supports is emphasized throughout, with participants encouraged to reach out to trusted individuals or groups for support. Participants can also select from a menu of potential personal change goals (e.g., autonomy) and learn about specific topics (e.g., obtaining a restraining order), which are presented as a series of empowerment videos, depicting diverse women sharing how they managed a specific IPV-related issue and the resulting positive outcome(s). SURE also includes one 10–15-minute telephone-delivered booster session, designed to bolster the effects of the intervention, one month after the initial online intervention. During the booster session, an interventionist reviews participants’ reasons for change, their goals and motivators, and discusses, problem-solves, and provides resources related to barriers to achieving goals. The interventionist also reinforces empowerment concepts and skills, goals for change, and links participants to potentially beneficial IPV resources. Randomly selected recordings of SURE booster sessions (*n* = 25) were rated for fidelity. Average adherence and competence ratings on a 6-point scale were excellent (*M(SD*)’s = 5.44(0.61) & 5.63(0.49), respectively).

The attention, time, and information-matched (i.e., both received some information on and resources for IPV) *control condition* is well-validated and has been successfully used in prior research with digital interventions (Ondersma et al. 2016; Tzilos et al. 2011; Tzilos Wernette et al. 2018; Zlotnick et al. 2019). Participants viewed videos of popular entertainers and television shows, followed by questions about participants’ preferences. During the booster session, interventionists reviewed participants’ ratings, initiated a more in-depth conversation about their preferences, and discussed other forms of entertainment that interested participants.

### Measures

All measures were administered at baseline, 6-week, 3, 6, and 12-month follow-up (FU).^3^

The eight-item *Woman Abuse Screening Tool* (WAST; Brown et al. 2000) was used to screen for past-year IPV. The WAST has correctly classified 100% of women who have not experienced IPV and 92% of women who have experienced IPV in a known group analysis (Schulz et al. 2014). Consistent with similar studies (e.g., Zlotnick et al. 2019), IPV status was considered positive if a woman scored 4 or more on the WAST.

The well-validated 30-item *Composite Abuse Scale* (CAS; Hegarty et al. 1999; Hegarty and Valpied 2013) was used to measure IPV severity. The CAS assesses physical, emotional, sexual, and combined severe abuse participants experienced over the last year at baseline, and since the last assessment at follow-up. The CAS has demonstrated good psychometric properties (Hegarty et al. 2005), and had excellent internal validity in the current study (*α* = 0.93).

The nine-item *Quality of Life in Neurological Disorders (Neuro-QoL)* scale for positive affect and well-being (National Institute of Neurological Disorders and Stroke 2015) was used to assess aspects of a person’s life related to their sense of well-being, life satisfaction, and/or overall sense of purpose and meaning. This computerized adaptive self-report measure is intended for use in clinical trials and has demonstrated sufficient reliability, internal consistency, and concurrent validity (Salsman et al. 2013), as well as internal consistency in the current study (*α* = 0.94).

The four-item emotional support bank from the *Patient-Reported Outcomes Measurement Information System (PROMIS©*; Cella et al. 2010; Hahn et al. 2014) was used to measure perceived emotional support. PROMIS*©* is a National Institutes of Health Roadmap initiative that provides precise, reliable, valid, and standardized questionnaires measuring patient-reported outcomes across physical, mental, and social health (Cella et al. 2010). The PROMIS*©* demonstrated excellent internal consistency in this study (*α* = 0.93).

### Procedures

All procedures were approved by the Women and Infants Hospital (Protocol# 12285109-69) and the University of Michigan Medical (Protocol# HUM00166275) IRBs following the Common Rule outlined in 45 CFR46. Best practice IPV safety procedures (e.g., safety planning during interviews, assuming the abusive partner may have access) were used throughout the study (e.g., Anderson et al. 2017).

Participants were recruited through two clinical sites in the Michigan and Rhode Island that provide mental health treatment specifically to perinatal women. Research assistants (RAs) identified a pool of potential participants using the hospital’s electronic medical system. RAs reached out to potential participants via phone, text, WhatsApp, and/or email to ask if they would participate in a brief health survey over the phone. Additional recruitment procedures included posting flyers in locations serving our target population (e.g., WIC offices), advertisements on social media (e.g., Instagram) targeting the two study states, and having obstetric clinics provide flyers to potential participants. In the latter cases, the RA reached out to potential participants who expressed an interest in the study via the same methods described above. Interested participants provided verbal consent and were administered the screener over the phone, which included the WAST, and assessed whether they received mental health services during pregnancy or post-partum. Participants who met eligibility criteria were informed of the nature of the study, asked if comfortable using the internet, as well as a series of questions designed to reduce the risks associated with participants remotely viewing an online IPV-focused intervention (e.g., Danger Assessment-5 (Messing et al. 2017) and the Technology Assessment Questionnaire (Havron et al. 2019; see Johnson et al. 2022 for full protocol). An RA reviewed the consent form, which was sent in the body of an email, to participants who met the study criteria. Verbal consent was documented in a password-protected document. CIAS randomized participants to SURE or control after the baseline assessment. Participants in both conditions were offered one 40-min online session and one 10–15 min telephone booster session one month after completing the online session, as well as a list of IPV-related resources at each research contact.

Participants at the Northeast study site were offered online or in-person participation at the hospital to view the intervention on a tablet when safety concerns were identified during screening. Participants at the Midwest study site were only offered online participation when safe. To further maximize safety, RAs assisted online participants in creating a temporary email to use when accessing study materials. Eligible remote participants were emailed an individual URL to complete the intervention in a safe and private location. Participants who did not meet study criteria were offered IPV-related and mental health resources.

### Data collection

Data were collected via CIAS, with participants entering responses directly when the assessment was completed in person or via an emailable URL link, or research staff who were blind to participants’ intervention condition entering responses into CIAS if the assessment was delivered by phone. Data were converted to a CSV file for analysis by an unblinded independent statistician, which has been found not to influence findings (Ifaifel et al. 2023).

### Data analysis

Baseline demographics were compared between SURE and control to identify the balance between the intervention arms. Linear Mixed regression models (LMM) were explored to identify any difference between conditions over time with regard to our continuous primary (i.e., IPV severity) and secondary outcomes (i.e., quality of life and perceived emotional support).^4^ Group (intervention vs. control), a 5-category time variable (baseline, 6-week, 3-, 6- and 12-month follow-up), and group-by-time interaction were used as primary covariates in the model. A random subject intercept was used to account for within-subject clustering. All models were adjusted for age, ethnicity, whether IPV was from the current partner, pregnancy status, education level, employment status, marital status, and site. Residual analyses revealed skewness in the IPV variable, which was shifted by 1 and log-transformed before applying LMM.

## Results

### Demographic characteristics, participant flow, and safety

**Fig. 1:**
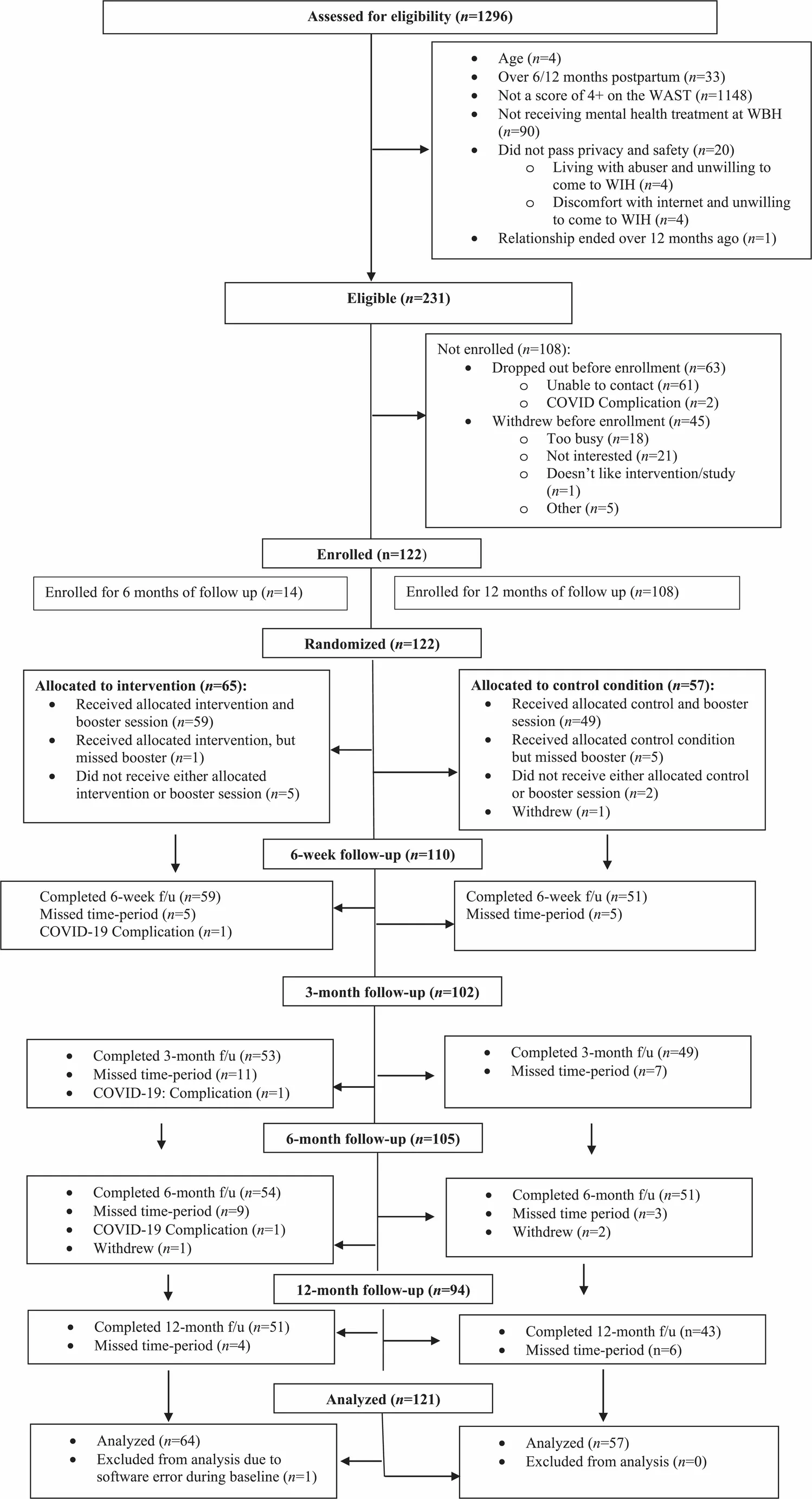
CONSORT diagram of participant flow throughout the study

Table 1 displays baseline participant characteristics. No significant demographic differences were found. Figure 1 depicts the flow of participants throughout the study. Overall retention rates were: 90.2% for 6-week FU, 83.6% for 3-month FU, 86.1% at 6-month FU, and 87% at 12-month FU. There were no study-related serious adverse events.

### Primary and secondary outcomes

**Fig. 2:**
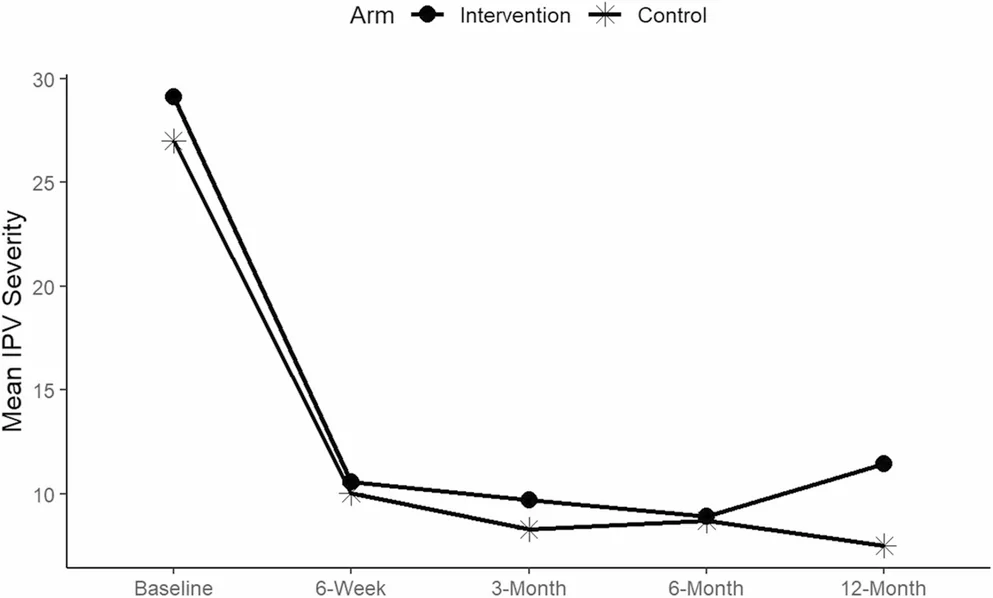
Mean IPV Severity by time and arm

There is a significant drop in IPV severity in both groups from baseline, which stayed significantly lower throughout the study period (Fig. 2). In the adjusted model, the estimated mean IPV severity values steadily decrease in SURE, with the six-month value being significantly lower than that at 3 months (*p* = .02) and is borderline significantly lower than that at six weeks (*p* = .06). However, scores go up and down in a random manner in the control arm. None of the pairwise differences in mean IPV severity between time-points post-randomization in the control arm are significantly different (Table 2(b)). Both education and employment status are significantly associated with the longitudinal IPV severity scores. Women with less than or equal to a high school degree or GED, and with a tech/trade degree and some college, experience significantly higher abuse than the women with a graduate or post-graduate degree. On the other hand, part-time and unemployed women score on average significantly lower in IPV compared to women who are employed full-time (Table 2(a)).

**Fig. 3:**
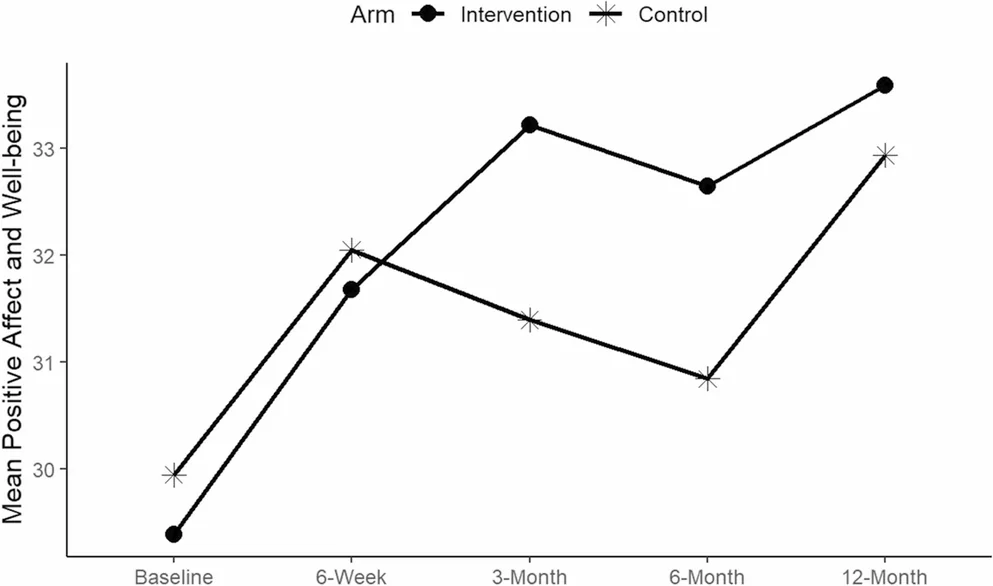
Mean positive affect and well-being by time and arm

For quality of life (QOL), SURE demonstrated a positive effect by retaining improvement throughout the study period. The control showed random fluctuation, and although there were some signs of improvement after 3 months, it never reached the mean QOL level in the SURE arm after six weeks (Fig. 3; Table 3(b)). No association with any other covariate was observed (Table 3(a)).

**Fig. 4:**
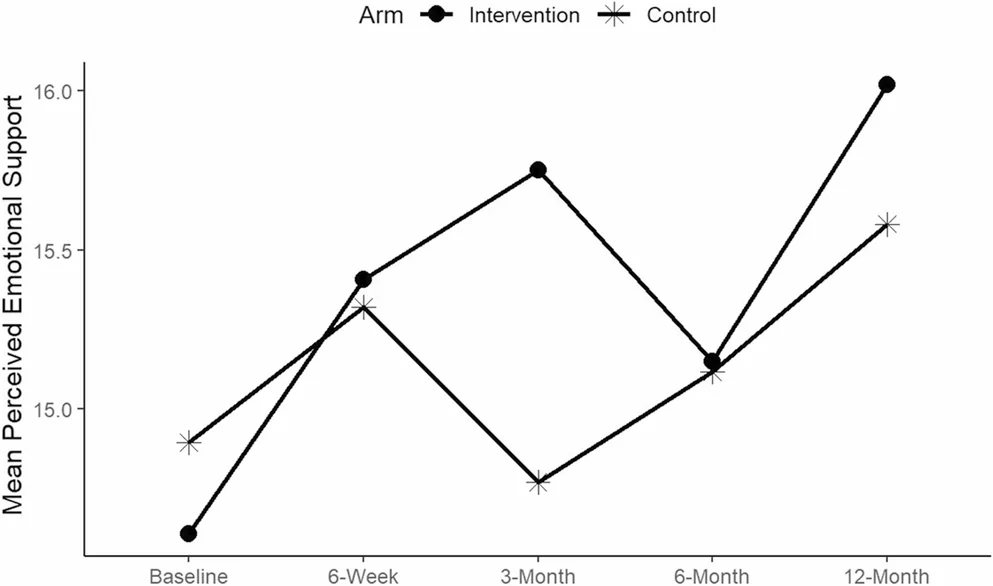
MEAN perceived emotional support by time and arm

While mean perceived emotional support values showed some fluctuation, the SURE group generally experienced significant improvement in perceived emotional support compared to baseline, while the control did not (Fig. 4; Table 4(b)). The improvement is greatest at the 3-month follow-up when the control experienced deterioration, resulting in a significant time-by-group difference in differences between 3 months and baseline (Table 4(a)).

Standardized effect sizes for time-by-group interactions at each follow-up timepoint are in Table 5.

### Discussion and conclusions

This study found that a brief online intervention, SURE, relative to a time, attention, and information-matched control, could be delivered safely and was associated with gains in positive affect and well-being and perceived emotional support in perinatal women who experienced recent IPV and recently engaged in mental health treatment. While this study did not find SURE to be associated with reduced IPV severity relative to control, both arms experienced reduced IPV severity across the 12-month follow-up. Participants in both conditions received IPV screening and referrals to IPV-related resources, an approach consistent with the recommendations of the US Preventitve Services Task Force (2025). Aligned with SUREs empowerment approach and focus on connecting women with both formal and informal support resources, SURE participants compared to those in the control condition showed improved well-being and perceived emotional support, providing further evidence that intervention beyond screening and referral can be beneficial to this vulnerable population (Mercier et al. 2024).

Our finding of no intervention effect for IPV severity is not surprising, given that women do not have control over whether their partner chooses to abuse them (Sullivan 2011). It could be more important for women to experience greater positive emotions and well-being and feel emotionally supported, both protective factors that can help women access important resources and better navigate their experiences of IPV. While research is needed to determine the mechanism through which SURE impacts perceived emotional support and well-being, SURE is consistent with empowerment-based interventions that have demonstrated efficacy in IPV-related outcomes (e.g., Johnson et al. 2020b).

### Strengths and limitations

Strengths of this study include the RCT design, diverse sample across multiple sites, inclusion of safety measures for online access, and use of standardized measures with strong psychometric properties. The retention rates across follow-up were excellent (i.e., 87% at 12-month follow-up). Weaknesses include the lack of an active control condition and the exclusion of mental health and other relevant outcomes (e.g., resource utilization). Additionally, our sample was relatively educated and financially advantaged; findings may not generalize to other IPV populations. A more intensive dose of the intervention, as opposed to a single session and a brief booster session, might be needed to impact IPV severity in perinatal women. Our sample size fell slightly short of our target sample size of 148 (Johnson et al. 2020a), which was determined assuming certain detectable effect sizes and variability. However, we were not quite underpowered. We demonstrated significant pairwise time differences in the primary outcome in both the intervention and control arms initially and in the intervention arm between certain post-randomization time points. Such differences were more prominent in certain secondary outcomes.

### Conclusions

Future research is needed to evaluate SURE in different settings and determine its efficacy with non-perinatal women. While several empirically supported, technology-based interventions targeting IPV exist (e.g., Emezue et al. 2022), to our knowledge, this is the first study to demonstrate the efficacy of a brief, online intervention for this unique population (Jahanfar et al. 2014; Hameed et al. 2021). This study found SURE to have a meaningful impact on perinatal women who have experienced IPV, improving both their psychological well-being and an important protective factor (i.e., emotional support) of mental health difficulties and revictimization (Goodman and Smyth 2011). SURE is easily accessible, brief, and low-cost, and can be easily implemented into diverse settings. Thus, implementation of SURE has the potential to have a substantial impact on the overall health and well-being of perinatal women who have experienced IPV.

**Table 1:** Study demographics by intervention arm*

Variables	Overall	Intervention arm		*P*-value
		SURE	Control	
Current Age (N, mean, SD)	122, 30.1, 6.2	65, 30.8, 6.3	57, 29.3, 6.0	0.2
Ethnicity (N, %)				0.7
Hispanic	25, 20.5	12, 18.5	13, 22.8	
Non-Hispanic	97, 79.5	53, 81.5	44, 77.2	
Race (N, %)				0.2
Caucasian/White	78, 63.9	45, 69.2	33, 57.9	
Black	29, 23.8	13, 20.0	16, 28.1	
Asian	1, 0.8	1, 1.5	0, 0.0	
Native American or Native Alaskan	2, 1.6	2, 3.1	0, 0.0	
Bi-Racial or Multi-Ethnic	6, 4.9	3, 4.6	3, 5.3	
Other	6, 4.9	1, 1.5	5, 8.8	
Marital Status (N, %)				0.5
Married	36, 29.8	22, 34.4	14, 24.6	
Separated	7, 5.8	4, 6.3	3, 5.3	
Divorced	6, 5.0	3, 4.7	3, 5.3	
Single, no relationship	34, 28.1	19, 29.7	15, 26.3	
Single, in a relationship	37, 30.6	15, 23.4	22, 38.6	
Single, same sex partner	1, 0.8	1, 1.6	0, 0.0	
Employment Status (N, %)				0.5
Full time	49, 40.5	24, 37.5	25, 43.9	
Part time	18, 14.9	7, 10.9	11, 19.3	
Student	6, 5.0	3, 4.7	3, 5.3	
Housewife	15, 12.4	9, 14.1	6, 10.5	
Unemployed	33, 27.3	21, 32.8	12, 21.1	
Education (N, %)				0.4
Less than HS	3, 2.5	2, 3.1	1, 1.8	
HS/GED	23, 19.0	11, 17.2	12, 21.1	
Tech/Trade school	5, 4.1	4, 6.3	1, 1.8	
Some Colleges	39, 32.2	16, 25.0	23, 40.4	
College Graduate	25, 20.7	15, 23.4	10, 17.5	
Postgraduate	26, 21.5	16, 25.0	10, 17.5	
Family Income (N, %)				0.5
I receive public assistance.	19, 16.2	13, 21.0	6, 10.9	
Less than $10,000	9, 7.7	3, 4.8	6, 10.9	
19,999	11, 9.4	6, 9.7	5, 9.1	
29,999	8, 6.8	3, 4.8	5, 9.1	
39,999	17, 14.5	7, 11.3	10, 18.2	
49,999	8, 6.8	5, 8.1	3, 5.5	
$50,000 +	45, 38.5	25, 40.3	20, 36.4	
# children (N, %)				0.5
0	13, 10.7	6, 9.4	7, 12.3	
1	47, 38.8	24, 37.5	23, 40.4	
2	33, 27.3	21, 32.8	12, 21.1	
3 or more	28, 23.1	13, 20.3	15, 26.3	
Pregnant (N, %)				0.8
No	91, 75.2	47, 73.4	44, 77.2	
Yes	30, 24.8	17, 26.6	13, 22.8	
IPV from Current Partner (N, %)				0.7
No	46, 38.0	26, 40.6	20, 35.1	
Yes	75, 62.0	38, 59.4	37, 64.9	
Site (N, %)				0.5
UM	65, 53.3	37, 56.9	28, 49.1	
WIH	57, 46.7	28, 43.1	29, 50.9	
Baseline CAS Total Score				0.5
(N, mean, SD)	121, 28.1, 20.7	64, 29.1, 20.9	57, 27.0, 20.7	
(Median, Q1, Q3)	24, 12, 40	24, 12, 45	24, 11, 34	

*Baseline demographics were compared between the SURE and control arms using 2-tailed *t* tests, Wilcoxon rank-sum tests, chi-square tests, as appropriate

**Table 2:** (a) LMM-based estimates for IPV-Severity. (b) Pairwise comparisons of LMM-based estimated means for the IPV-Severity

Parameter		Estimate		Standard error		*P*-value
Age at Enrollment		0.0123		0.0183		0.5040
Ethnicity (ref: Hispanic/Latina)		0.1189		0.2367		0.6158
IPV From Current Partner (ref: No)		-0.1910		0.3992		0.6326
Pregnancy Status (ref: No)		-0.1728		0.2278		0.4487
Education (ref: Graduate/Postgraduate)						
Less than HS		0.7337		0.2966		0.0138
Tech/Trade/Some College		0.5766		0.2446		0.0189
Employment (ref: Full time)						
Part time		-0.5673		0.2733		0.0386
Not employed		-0.5073		0.2111		0.0167
Marital Status (ref: Currently Single)						
Currently Married		0.4729		0.4149		0.2550
Single in relationship		0.3365		0.4118		0.4143
Site (ref: WIH)		-0.0492		0.2051		0.8107
Arm (Ref: Control)		0.1072		0.2264		0.6360
Time (Ref: Baseline)						
Intervention: 6-Week		-1.1685		0.1609		<.0001
Intervention: 3-Month		-1.0913		0.1678		<.0001
Intervention: 6-Month		-1.4887		0.1667		<.0001
Intervention: 12-Month		-1.2883		0.1675		<.0001
Control: 6-Week		-1.4024		0.1719		<.0001
Control: 3-Month		-1.4915		0.1744		<.0001
Control: 6-Month		-1.4653		0.1709		<.0001
Control: 12-Month		-1.6359		0.1806		<.0001
Arm * Time						
6-Week		0.2339		0.2354		0.3209
3-Month		0.4001		0.2419		0.0988
6-Month		-0.0234		0.2387		0.9220
12-Month		0.3476		0.2463		0.1590
Comparison	Intervention			Control		
	Mean Difference (time2-time1)	Standard Error	*P*-value	Mean Difference (time2-time1)	Standard Error	*P*-value
Baseline vs. 6-Week	-1.1685	0.1609	<.0001	-1.4024	0.1719	<.0001
Baseline vs. 3-Month	-1.0913	0.1678	<.0001	-1.4915	0.1744	<.0001
Baseline vs. 6-Month	-1.4887	0.1667	<.0001	-1.4653	0.1709	<.0001
Baseline vs. 12-Month	-1.2883	0.1675	<.0001	-1.6359	0.1806	<.0001
6-Week vs. 3-Month	0.0772	0.1698	0.6498	-0.0890	0.1784	0.6179
6-Week vs. 6-Month	-0.3202	0.1687	0.0584	-0.0629		
6-Week vs. 12-Month	-0.1198	0.1711	0.4842	-0.2335		
3-Month vs. 6-Month	-0.3974	0.1743	0.0231	0.0261		
3-Month vs. 12-Month	-0.1970	0.1768	0.2658	-0.1445		
6-Month vs. 12-Month	0.2004	0.1761	0.2558	-0.1706		

**Table 3:** (a) LMM-based estimates for positive affect and well-being. (b) Pairwise comparisons of LMM-based estimated means for positive affect and well-being

Parameter		Estimate		Standard error		*P*-value
Age at Enrollment		-0.0049		0.1095		0.9647
Ethnicity (ref: Hispanic/Latina)		0.0997		1.4195		0.9441
IPV From Current Partner (ref: No)		-0.1798		2.3679		0.9395
Pregnancy Status (ref: No)		-0.0099		1.3619		0.9942
Education (ref: Graduate/Postgraduate)						
Less than HS		-1.6129		1.7745		0.3639
Tech/Trade/Some College		0.4607		1.4673		0.7537
Employment (ref: Full time)						
Part time		0.0144		1.6396		0.9930
Not employed		0.0508		1.2666		0.9680
Marital Status (ref: Currently Single)						
Currently Married		-1.3968		2.4567		0.5700
Single in relationship		-0.6599		2.4476		0.7876
Site (ref: WIH)		1.1581		1.2296		0.3468
Arm (Ref: Control)		-0.6139		1.2621		0.6269
Time (Ref: Baseline)						
Intervention: 6-Week		1.9511		0.7725		0.0119
Intervention: 3-Month		3.3903		0.8063		<.0001
Intervention: 6-Month		2.8193		0.8009		0.0005
Intervention: 12-Month		3.9416		0.8047		<.0001
Control: 6-Week		1.9910		0.8256		0.0163
Control: 3-Month		1.5999		0.8375		0.0568
Control: 6-Month		0.9600		0.8207		0.2428
Control: 12-Month		3.1466		0.8676		0.0003
Arm * Time						
6-Week		-0.0399		1.1304		0.9718
3-Month		1.7904		1.1621		0.1242
6-Month		1.8594		1.1466		0.1057
12-Month		0.7950		1.1833		0.5021
Comparison	Intervention			Control		
	Mean Difference (time2-time1)	Standard Error	*P*-value	Mean Difference (time2-time1)	Standard Error	*P*-value
Baseline vs. 6-Week	1.9511	0.7725	0.0119	1.991	0.8256	0.0163
Baseline vs. 3-Month	3.3903	0.8063	<.0001	1.5999	0.8375	0.0568
Baseline vs. 6-Month	2.8193	0.8009	0.0005	0.96	0.8207	0.2428
Baseline vs. 12-Month	3.9416	0.8047	<.0001	3.1466	0.8676	0.0003
6-Week vs. 3-Month	1.4392	0.8144	0.078	-0.3912	0.8554	0.6477
6-Week vs. 6-Month	0.8682	0.8091	0.2839	-1.031	0.8466	0.224
6-Week vs. 12-Month	1.9905	0.8217	0.0159	1.1556	0.8917	0.1957
3-Month vs. 6-Month	-0.5709	0.8358	0.495	-0.6399	0.8521	0.4531
3-Month vs. 12-Month	0.5514	0.8488	0.5163	1.5468	0.8994	0.0863
6-Month vs. 12-Month	1.1223	0.8456	0.1852	2.1866	0.8829	0.0137

**Table 4:** (a) LMM based estimates for perceived emotional support. (b) Pairwise comparisons of LMM-based estimated means for perceived emotional support

Parameter		Estimate		Standard error		*P*-value
Age at Enrollment		0.0074		0.0676		0.9126
Ethnicity (ref: Hispanic/Latina)		0.3106		0.8792		0.7241
IPV From Current Partner (ref: No)		2.1578		1.4552		0.1389
Pregnancy Status (ref: No)		0.8877		0.8418		0.2923
Education (ref: Graduate/Postgraduate)						
Less than HS		-1.5196		1.0971		0.1668
Tech/Trade/Some College		-1.2017		0.9087		0.1868
Employment (ref: Full time)						
Part time		0.3373		1.0154		0.7399
Not employed		0.5347		0.7845		0.4959
Marital Status (ref: Currently Single)						
Currently Married		-2.8288		1.5080		0.0614
Single in relationship		-1.2982		1.5065		0.3894
Site (ref: WIH)		0.9076		0.7613		0.2339
Arm (Ref: Control)		-0.4252		0.7461		0.5691
Time (Ref: Baseline)						
Intervention: 6-Week		0.7533		0.3944		0.0569
Intervention: 3-Month		1.1233		0.4118		0.0067
Intervention: 6-Month		0.5218		0.4091		0.2028
Intervention: 12-Month		1.2506		0.4108		0.0025
Control: 6-Week		0.3780		0.4215		0.3703
Control: 3-Month		-0.1237		0.4277		0.7726
Control: 6-Month		0.0829		0.4190		0.8432
Control: 12-Month		0.6812		0.4431		0.1250
Arm * Time						
6-Week		0.3753		0.5771		0.5159
3-Month		1.2470		0.5935		0.0363
6-Month		0.4389		0.5855		0.4540
12-Month		0.5694		0.6042		0.3466
Comparison	Intervention			Control		
	Mean Difference (time2-time1)	Standard Error	*P*-value	Mean Difference (time2-time1)	Standard Error	*P*-value
Baseline vs. 6-Week	0.7533	0.3944	0.0569	0.3780	0.4215	0.3703
Baseline vs. 3-Month	1.1233	0.4118	0.0067	-0.1237	0.4277	0.7726
Baseline vs. 6-Month	0.5218	0.4091	0.2028	0.0829	0.4190	0.8432
Baseline vs. 12-Month	1.2506	0.4108	0.0025	0.6812	0.4431	0.1250
6-Week vs. 3-Month	0.3700	0.4155	0.3736	-0.5017	0.4363	0.2510
6-Week vs. 6-Month	-0.2315	0.4127	0.5752	-0.2951	0.4320	0.4950
6-Week vs. 12-Month	0.4973	0.4194	0.2364	0.3032	0.4552	0.5057
3-Month vs. 6-Month	-0.6015	0.4263	0.1591	0.2066	0.4346	0.6348
3-Month vs. 12-Month	0.1273	0.4333	0.7691	0.8049	0.4591	0.0803
6-Month vs. 12-Month	0.7288	0.4317	0.0922	0.5983	0.4505	0.1849

**Table 5:** Standardized effect sizes for differences between SURE and control from baseline at each study follow-up point

Outcomes	Time point	Standardized effect sizes for time-by-group interaction
IPV-Severity	6-Week	0.99
	3-Month	1.65
	6-Month	-0.10
	12-Month	1.41
Positive Affect and Well-being	6-Week	-0.04
	3-Month	1.54
	6-Month	1.62
	12-Month	0.67
Perceived Emotional Support	6-Week	0.65
	3-Month	2.10
	6-Month	0.75
	12-Month	0.94

## Data Availability

This study was initiated before the normative practice of data depositories, and therefore, participants did not consent to have their data publicly available. Data are available by request to the last author.

## Footnotes

1 Original criterion was up to 6 months postpartum (Johnson et al. 2020a) but was extended to 12 months to expand pool of participants.

2 Original criterion was that participants were seeking treatment in one of the two sites perinatal mental health clinic; this criterion was adapted during COVID.

3 A small subset of participants (*n* = 14) was enrolled for six, as opposed to 12-month follow-up, due to insufficient RA resources to follow participants for the full 12 months (Fig. 1).

4 Since there are no time-varying covariate, no additional adjustment is made for handing missing outcome data, since LMM provides valid results even under Missing at Random (MAR) assumption. LMM uses all available data, and therefore supports intent-to-treat principles.
