# Correction to: An online intervention targeting intimate partner violence in perinatal women with recent mental health care utilization: a multisite randomized clinical trial

**DOI:** 10.1007/s00737-026-01775-2

**Published:** 2026-09-30

**Authors:** Dawn M. Johnson, Ananda Sen, Dongru Chen, Kristina Countryman, Maria Muzik, Briana Joseph, Golfo Tzilos Wernette, Caron Zlotnick

**Affiliations:** 1 University of Akron, Akron, USA; 2 University of Michigan, Ann Arbor, USA; 3 Women & Infants Hospital, Providence, USA; 4 University of Cape Town, Cape Town, South Africa

**Correction to: Archives of Women's Mental Health (2026) 29:68**

**https://doi.org/10.1007/s00737-026-01703-4**

In the original article, "An online intervention targeting intimate partner violence in perinatal women with recent mental health care utilization: a multisite randomized clinical trial", the name of one of the senior authors was misspelled.

- **Published form (with error):** Caron Zlontick
- **Corrected form:** Caron Zlotnick

The original article has been corrected.

